# Supplementary figures and images for: Impact of dormancy periods on some physiological and biochemical indices of potato tubers
Source: PeerJ. 2023 Aug 28;11:e15923. doi: 10.7717/peerj.15923 (PMC10470445; doi:10.7717/peerj.15923)

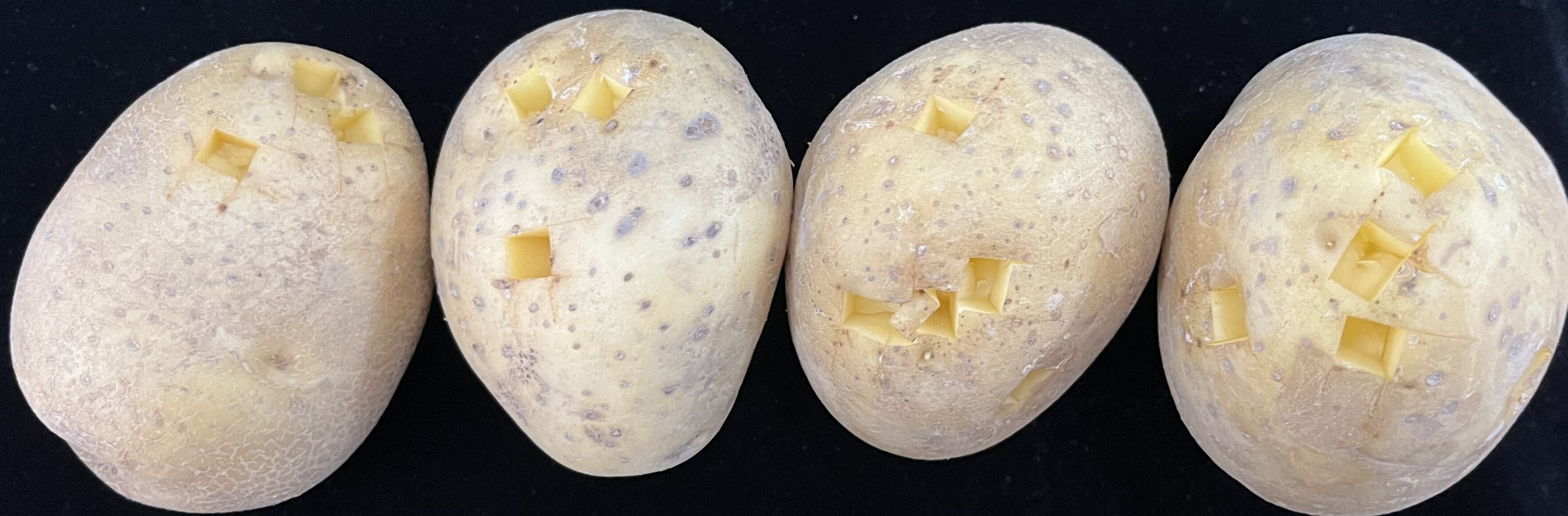

Supplement: Supplemental Information 2 [file peerj-11-15923-s002.pdf]

**SDV: Yunsu 306**

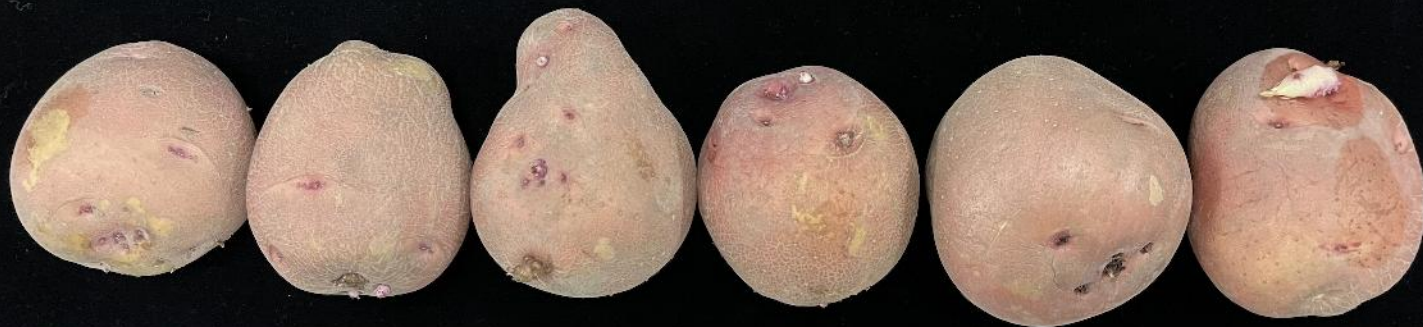

**MDV: Yunsu 304**

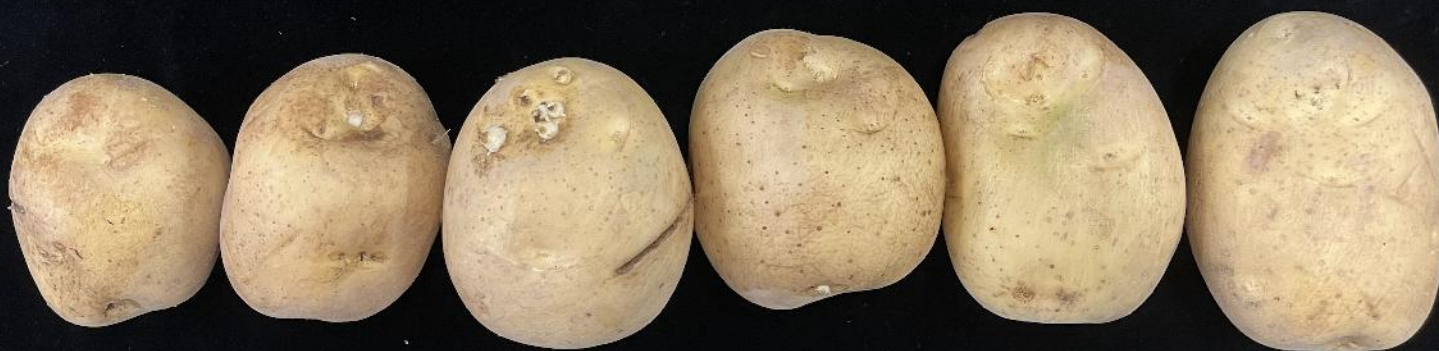

**LDV: Yunsu108**

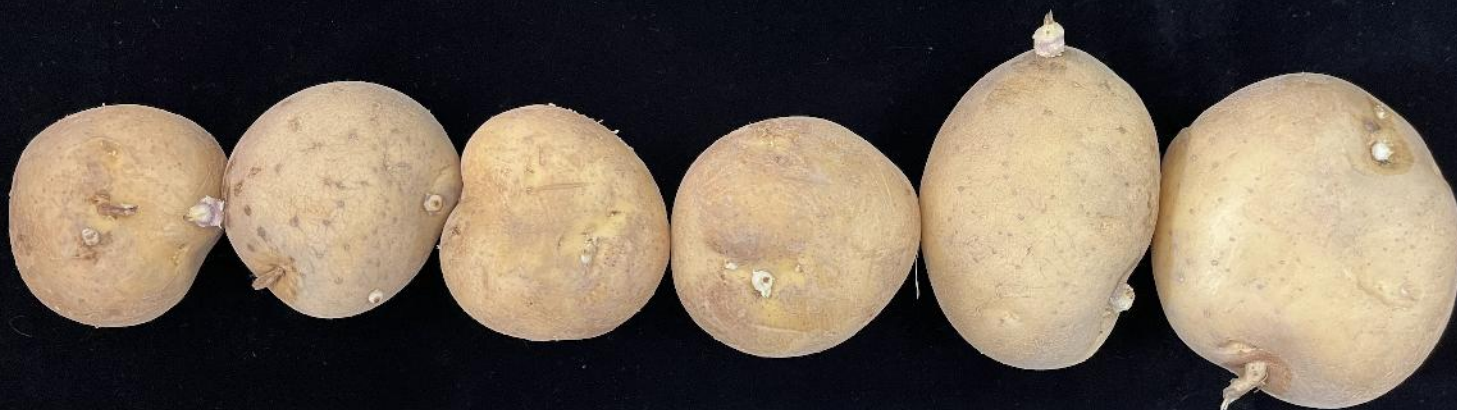

Supplement: Supplemental Information 3 [file peerj-11-15923-s003.pdf]
